# Preventing peripheral intravenous catheter failure by reducing mechanical irritation

**DOI:** 10.1038/s41598-019-56873-2

**Published:** 2020-01-31

**Authors:** Toshiaki Takahashi, Ryoko Murayama, Mari Abe-Doi, Maki Miyahara-Kaneko, Chiho Kanno, Miwa Nakamura, Mariko Mizuno, Chieko Komiyama, Hiromi Sanada

**Affiliations:** 1 Department of Life Support Technology (Molten), Graduate School of Medicine, The University of Tokyo, Tokyo, Japan; 2 Department of Advanced Nursing Technology, Graduate School of Medicine, The University of Tokyo, Tokyo, Japan; 3 Global Nursing Research Center, Graduate School of Medicine, The University of Tokyo, Tokyo, Japan; 4 Department of Gerontological Nursing/Wound Care Management, Graduate School of Medicine, The University of Tokyo, Tokyo, Japan; 5 Department of Nursing, The University of Tokyo Hospital, Tokyo, Japan

**Keywords:** Ultrasonography, Outcomes research

## Abstract

Peripheral intravenous catheter failure is a significant concern in the clinical setting. We investigated the effectiveness of care protocols, including an ultrasonographic “pre-scan” for selecting a large-diameter vein before catheterization, a “post-scan” for confirming the catheter tip position after catheterization with ultrasonography, and the use of a flexible polyurethane catheter to reduce the mechanical irritation that contributes to the incidence of catheter failure. This intervention study was a non-randomized controlled trial to investigate the effectiveness of the abovementioned care protocols, the effects of which were compared to the outcomes in the control group, which received conventional care. For both groups, participants were selected from patients in two wards at the University of Tokyo in Japan between July and November 2017. Inverse probability score-based weighted methods (IPW) using propensity score were used to estimate the effectiveness of care protocols. The primary outcome was catheter failure, which was defined as accidental and unplanned catheter removal. We used Kaplan-Meier survival curves to compare rates of time until catheter failure. We analysed 189 and 233 catheters in the intervention and control groups, respectively. In the control group, 68 catheters (29.2%) were determined to have failed, whereas, in the intervention group, only 21 catheters (11.1%) failed. There was a significant difference between each group regarding the ratio of catheter failure adjusted according to IPW (*p* = 0.003). The relative risk reduction of the intervention for catheter failure was 0.60 (95% CI: 0.47–0.71). Care protocols, including assessment of vein diameter, vein depth, and catheter tip location using ultrasound examination for reducing mechanical irritation is a promising method to reduce catheter failure incidence.

## Introduction

### Catheter failure

Most patients require at least one peripheral vascular device for delivering intravenous fluids and medications during their hospital stay. A peripheral intravenous catheter (PIVC) is commonly used. Recent studies reported that >70% of patients in acute care hospitals use PIVCs^1–3^. Additionally, >25% of PIVCs are reportedly accidentally removed, which is termed catheter failure^3–5^. In a study conducted over 2 months at a university hospital in Tokyo, Japan, 5,316 catheters from 2,442 patients were studied; the rate of catheter removal due to catheter failure was 18.8%^6^. Catheter failure is associated with signs and symptoms such as erythema, swelling, induration, bleeding, pain, and insufficient dripping^5,7,8^. These symptoms negatively affect patient comfort and treatment, eventually making it difficult to continue intravenous therapy^1,9^. In such cases, catheters need to be replaced, which makes the patient uncomfortable, increases labour, and drives up costs^10–12^. Therefore, it is important for both patients and healthcare providers to prevent catheter failure in PIVC.

### Preventing catheter failure

The risk factors for various PIVC complications are known, but healthcare providers cannot adequately prevent them (e.g., phlebitis and infiltration)^13–15^. Although equipment, technology, and education are constantly improving, these problems still exist, causing issues with patient care such as feelings of discomfort and deciding to discontinue treatment. The current preventive measures are insufficient; novel measures are needed. Previous studies suggested that mechanical irritation was an important factor in catheter failure^7,16^. Therefore, we focused on mechanical irritation. Weiss *et al*. have reported many effects on vascular inflammation and thrombus formation caused by catheter mechanical stimulation^17–19^. Our laboratory also reported that PIVC tip contact with the vessel wall was associated with subcutaneous oedema^20,21^. These studies suggested that the patient’s posture during insertion and fixation could affect thrombus and oedema formation, as mechanical irritation affects the veins and subcutaneous tissue^16,19–21^.

Next, we analysed the relationship between vein diameter and catheter failure. Our laboratory analysed vein diameter and calculated the ratios of the vein diameter to catheter gauge under the same conditions as those described previously^22^. The relationship between these ratios and infiltration was assessed to determine a cut-off point. The mean ratio of vein diameter in the infiltration group was significantly smaller than in the no-infiltration group (*p* < 0.01), and the ratio was an independent risk factor on multivariable analysis. The ratio of 3.3 times was determined to be the cut-off point enabling healthcare providers to identify veins appropriately. For example, if a 22-gauge catheter is selected, a site where vein diameter exceeds 3.0 mm is suitable.

Last, the relationship of the catheter material, angle, and failure was analysed using ultrasonography (US)^23^. This study suggested that a polyurethane catheter was effective in preventing catheter failure due to its softness. Our previous study on the risk factors for phlebitis reported an association between the use of polyurethane catheters and a 30%-50% reduction in phlebitis occurrence compared to after using polytetrafluorethylene (Teflon^®^) catheters^24–26^. Therefore, in this study we also included the use of a polyurethane catheter.

Our laboratory focused on damage to the vein by mechanical irritation including three parameters, which were the appropriate catheter tip position, vein diameter, and catheter material.

### Care bundle approach for preventing catheter failure

Despite numerous randomized controlled trials (RCTs) on PIVC worldwide^27^ catheter failure remains unresolved. This may be because intravenous therapy involves multiple steps, including site selection, catheterization, and fixation. Performing one or some of these processes optimally is insufficient. Rather than focusing on the outcomes of each process, it is essential to introduce the outcome of all processes simultaneously. The approach for such interventions is akin to the care bundle approach, which is employed in clinical settings for controlling infection and preventing ventilator-associated pneumonia^28–30^. A care bundle is defined as a “set of processes, generally three to five, that are proven by RCTs and are performed as a whole to obtain optimal outcomes”^28^. Using a bundle approach to develop care practices for controlling infection and preventing ventilator-associated pneumonia yields positive clinical outcomes^29^. In PIVC infection management, performing multiple intervention has been shown to yield certain positive outcomes^31^, but the care bundle approach has not been reported to prevent catheter failure. In our laboratory, several studies have yielded implications for clinical care. These studies provide evidence for processes such as appropriate selection of puncture site and device and fixation method. We believe that conducting the intervention using bundled concepts is possible, especially when focused on preventing catheter failure by reducing mechanical irritation. In this study, we bundled three points extracted from the analysis of our previous research and called it a “care bundle”, that is, a “pre-scan” for selecting a large vein diameter before catheterization, “post-scan” for confirming the catheter tip position after catheterization, and “use of polyurethane catheter,” which is a catheter type with more flexibility.

### Aim

This study aimed to establish and evaluate a three-point care bundle intervention method to prevent catheter failure.

## Results

### Study population

**Figure 1:**
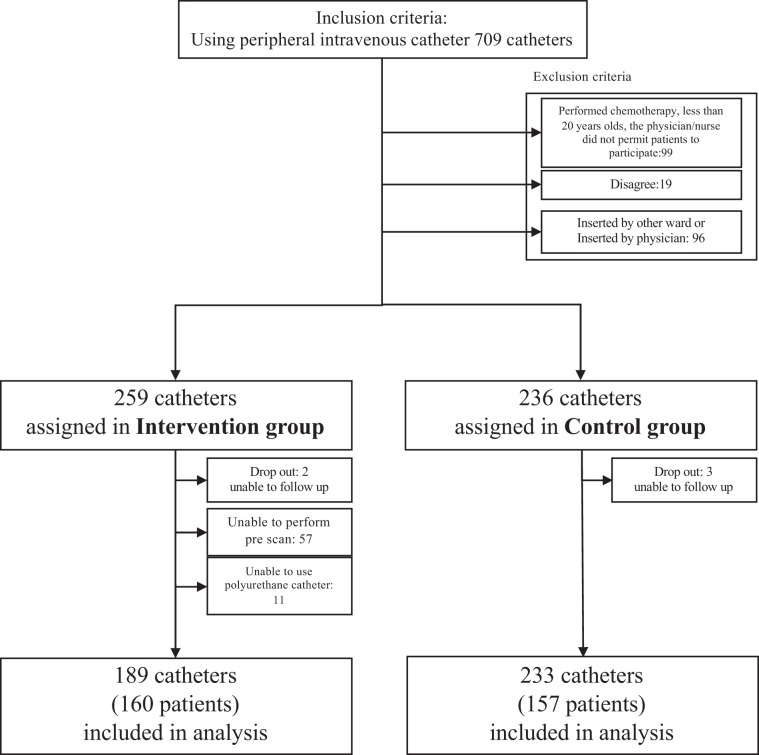
Flowchart of the subjects.

Among 706 eligible catheters, 119 were excluded per the exclusion criteria. A total of 272 and 315 catheters were finally allocated in the interventional and control groups, respectively. In the intervention group, 2, 17, 57, and 11 catheters were excluded due to patient dropout from lack of follow up by the patient moving to another ward, patient condition, inability to perform pre-scan in the patient, and inability to use a polyurethane catheter in the patient, respectively. In the control group, 3 and 79 catheters were excluded due to patient dropout. Finally, we analysed 189 catheter insertions using a pre-scan and post-scan using the appropriate catheter in the intervention group, and 233 catheterizations in the control group (Fig. 1). In this study, no adverse events such as unexpected catheter removal or skin problems related to the intervention occurred. The characteristics of participants unadjusted and adjusted using propensity scores are also shown in Table 1. There were significant differences between the intervention and control groups in present illness (tumour), CRP, albumin, dressing films, level of nurse experience, number of catheterizations catheterization times”, and hyperosmotic solutions. These variables showed differences between groups before adjustment but exhibited no difference between groups after adjustment except for number of catheterizations catheterization times. Regarding the number of catheterizations catheterization times, although the difference between the intervention and control groups was less, significant differences between groups remained.

**Table 1:** Characteristics of participants unadjusted and adjusted using IPW (weighted by inverse probability of propensity score).

	Unadjusted					Adjusted				
	Control group		Intervention group		*p-*Value	Control group		Intervention group		*p-*Value
	233		189							
Sex					0.39					0.48
Male	162	(69.5)	124	(65.6)			(65.9)		(68.9)	
Age (years)	66.4	± 15.9	65.2	± 14.3	0.40	65.7	± 15.9	64.4	± 14.3	0.21
BMI	23.1	± 3.4	22.9	± 4.1	0.51	23.1	± 3.3	22.8	± 4.3	0.36
Present illness (tumour)										
Tumour	96	(41.2)	121	(64.0)	0.00*		(47.4)		(53.0)	0.11^†^
Present illness (each organ)					0.59					0.51
Gastroenterology	62	(26.6)	50	(26.5)			(25.3)		(28.6)	
Hepatobiliary Pancreatic	153	(65.7)	129	(68.3)			(68.4)		(66.1)	
Other	18	(7.7)	10	(5.3)			(6.3)		(5.3)	
Diabetes	39	(16.7)	44	(23.3)	0.09		(19.5)		(19.8)	0.93
Oral medicine (anticoagulant)	35	(15.0)	26	(13.8)	0.76		(13.3)		(12.6)	0.79
Oral medicine (steroid)	8	(3.4)	12	(6.3)	0.21		(5.3)		(4.8)	0.75
Chemotherapy	10	(4.3)	10	(5.3)	0.63		(5.0)		(4.8)	0.87
Radiation	1	(0.4)	1	(0.5)	0.88		(0.3)		(0.2)	0.97
The level of need for nursing (Kangodo)					0.18					0.15
A1	1	(0.4)	2	(1.1)			(0.8)		(0.7)	
A2	4	(1.7)	2	(1.1)			(1.8)		(0.7)	
A3	4	(1.7)	2	(1.1)			(1.8)		(2.2)	
A4	1	(0.4)	0	(0.0)			(0.3)		(0.0)	
B1	19	(8.2)	13	(6.9)			(8.3)		(12.7)	
B2	5	(2.1)	8	(4.2)			(3.3)		(3.1)	
B3	108	(46.4)	70	(37.0)			(41.5)		(32.5)	
B4	51	(21.9)	38	(20.1)			(22.6)		(23.7)	
C1	0	(0.0)	0	(0.0)			(0.0)		(0.0)	
C2	0	(0.0)	0	(0.0)			(0.0)		(0.0)	
C3	2	(0.9)	5	(2.6)			(1.5)		(1.7)	
C4	38	(16.3)	49	(25.9)			(18.3)		(22.7)	
Blood examination										
C-reactive protein	2.81	± 4.7	1.08	± 2.5	0.00*	2.2	± 4.1	1.7	± 3.4	0.08^†^
Albumin	3.5	± 0.7	3.8	± 0.7	0.00*	3.6	± 0.7	3.5	± 1.7	0.19^†^
Platelet	21.8	± 10.9	20.4	± 9.2	0.20	21.2	± 11.2	21.0	± 9.3	0.75
Insertion side					0.70					0.74
Right	124	(53.2)	92	(48.7)			(49.4)		(48.2)	
Dressing films					0.03*					0.88^†^
IV®3000®	217	(93.1)	187	(98.9)			(95.5)		(95.7)	
Tegadearm®1648	16	(6.9)	2	(1.1)			(4.5)		(4.3)	
Level of nurse					0.00*					0.14^†^
Beginner	155	(66.5)	88	(46.6)			(60.9)		(54.2)	
Intermediate	34	(14.6)	82	(43.4)			(22.3)		(27.0)	
Expert	44	(18.9)	19	(10.1)			(16.8)		(18.9)	
Number of catheterizations					0.00*					0.01*
Once	123	(52.8)	155	(82.0)			(64.7)		(73.4)	
Twice	110	(47.2)	34	(18.0)			(35.3)		(26.6)	
Pharmacologic factor										
Hyperosmotic	53	(22.7)	21	(11.1)	0.00*		(18.8)		(20.0)	0.24†
Antibacterial	145	(62.2)	128	(67.7)	0.17		(64.2)		(60.6)	0.30
Lipid solution	4	(1.7)	2	(1.1)	0.53		(1.3)		(0.7)	0.44
Total rocking time (unused time)	37.3	± 43.5	45.4	± 37.2	0.05*	41.5	± 47.3	44.0	± 38.2	0.40

Data are expressed as n (%) ± SD. PIVC, peripheral intravenous catheterization; ND, no data

*p < 0.05; ^†^disappear of significant difference after adjustment.

Level of nurse experience; beginner, 0–100 PIVCs; intermediate, 101–800 PIVCs; expert, 801 + PIVCs.

### Incidence of catheter failure

The cumulative incidences of catheter failure before adjustment were 68 (29.2%) and 21 catheters (11.1%) in the control and intervention groups, respectively. Catheter failure per 1,000 catheter days was 89.5/1,000 and 35.0/1,000 catheter days in the control and intervention groups, respectively.

There was a significant difference between each group in terms of the ratio of catheter failure adjusted using IPW of propensity score (*p* = 0.003). The relative risk reduction (RRR) of the intervention for catheter failure was 0.60 (95% CI: 0.47 to 0.71) and the number needed to treat (NNT) was calculated as 6.04 (95% CI: 4.71 to 8.82) adjusted using IPW of propensity score. The same trend was obtained in the analysis by patient (Tab. S1, S2, S3).

**Figure 2:**
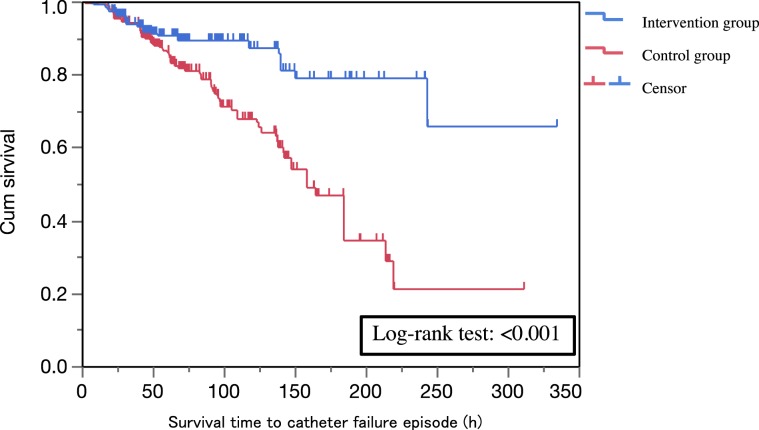
Kaplan-Meier survival curve of time weighted with inverse probability of propensity score. The censor was defined as “removal of catheter after completion of treatment required”.

Regarding survival analysis, the Kaplan-Meier curves are shown in Fig. 2. This figure shows a comparison of the intervention and control groups regarding survival time analysis adjusted using IPW of propensity score. There was a significant difference in the log-rank test (*p* < 0.001) between the two groups.

### Surrogate outcomes

The vein diameter, vein depth, and catheter tip position of the control and intervention groups were compared. The mean (SD) vein diameter in the control and intervention groups were 2.53 (0.70) and 2.75 (0.77) mm, respectively. The mean (SD) vein depth in the control and intervention groups were 2.21 (0.92) and 2.03 (0.81) mm, respectively. The proportion of appropriate catheter tip position in the control and intervention groups were 45.9% and 68.0%, respectively. Regarding vein diameter, there was a certain trend toward significant difference between the intervention and control groups (*p* = 0.004), and a trend was found in vein depth (*p* = 0.051). Regarding catheter tip position, the intervention group had higher rate of appropriate catheter tip position compared with the control group (*p* < 0.001).

### Exploration of risk factors

Both the intervention and control groups were included in the analysis (n = 422). Table 2 shows all univariate analyses. There were significant differences regarding present illness (tumour), CRP (mg/dL), albumin (mg/dL), number of catheterizations catheterization times, total unused time (locked time), insertion anatomical site, dominant vein, and hyperosmotic solution. Among these candidates, the multicollinearity was evaluated and a correlation was found between hyperosmotic solution and present illness (tumour) and number of catheterizations catheterization times. Moreover, correlation was found between dominant vein and insertion anatomical site and between CRP (mg/dL) and albumin (mg/dL). As a result, hyperosmotic solution, CRP (mg/dL), total unused time (locked time), and insertion anatomical site were entered the multivariate logistic regression. Then, we sequentially introduced groups of variables into the model. First, catheter variables (age, sex, BMI, and care bundle intervention) as confounding factors, then the use of a hyperosmotic” solution, CRP (mg/dL), total unused time (locked time), and insertion anatomical site. This hierarchical multiple logistic regression analysis was performed and illustrated in Table 3. As a result, care bundle intervention continued to show significant difference in all models (*p* < 0.01), hyperosmotic solution, CRP (mg/dL), and insertion anatomical site (compared with another site) also showed significant difference (*p* < 0.01, 0.04, and 0.01). Even if all the variables were input, Hosmer-Lemeshow test showed no significant difference.

**Table 2:** Characteristics of participants (n = 422) and univariate analysis results on catheter failure.

	Catheter failure (n = 89)	Complete to treat (n = 333)	*p*-Value
Sex (male)	55 (61.8)	231 (69.4)	<0.18
Age (years)	66.9 ± 18.6	65.6 ± 14.2	<0.48
BMI	23.5 ± 03.7	22.9 ± 03.7	<0.16
Present illness			
Tumour	33 (37.1)	184 (55.3)	<0.01*
Each organ			<0.86
Gastroenterology	24 (27.0)	088 (26.4)	
Hepatobiliary/pancreatic	58 (65.2)	224 (67.3)	
Others	070 (7.9)	0210 (6.3)	
Diabetes	16 (18.0)	067 (20.1)	<0.65
Oral medicine			
Anticoagulant	11 (12.4)	050 (15.0)	<0.53
Steroid	020 (2.2)	0180 (5.4)	<0.21
Chemotherapy	060 (6.7)	0140 (4.2)	<0.22
Radiation	000 (0.0)	0020 (0.6)	<0.46
Level of need for nursing (Kangodo)			<0.11
A1	000 (0.0)	003 0(0.9)	
A2	020 (2.2)	0040 (1.2)	
A3	020 (2.2)	0040 (1.2)	
A4	000 (0.0)	0010 (0.3)	
B1	10 (11.2)	022 0(6.6)	
B2	050 (5.6)	0080 (2.4)	
B3	43 (48.3)	135 (40.5)	
B4	17 (19.1)	072 (21.6)	
C1	000 (0.0)	0000 (0.0)	
C2	000 (0.0)	0000 (0.0)	
C3	000 (0.0)	0070 (2.1)	
C4	10 (11.2)	077 (23.1)	
Blood examination			
C-reactive protein (mg/dl)	03.5 ± 04.6	01.6 ± 03.7	<0.01*
Albumin (mg/dl)	03.4 ± 00.7	03.7 ± 00.7	<0.01*
Platelet (10^4^/μl)	22.7 ± 10.8	20.8 ± 10.0	<0.11
Insertion side			
Right	43 (48.3)	158 (47.4)	<0.88
Dressing films			<0.90
IV^®^3000^®^	040 (4.5)	0140 (4.2)	
Tegaderm^®^1648	85 (95.5)	319 (95.8)	
Level of nurse experience			<0.72
Beginner	48 (53.9)	195 (58.6)	
Intermediate	26 (29.2)	090 (27.0)	
Expert	15 (16.9)	048 (14.4)	
Number of catheterizations			<0.01*
First	44 (49.4)	234 (70.3)	
Second or more	45 (50.6)	099 (29.7)	
Total unused time (locked time)	31.3 ± 39.5	43.5 ± 41.3	<0.01*
Insertion anatomical site			<0.01*
Forearm	74 (83.1)	315 (94.6)	
Upper arm	060 (6.7)	0140 (4.2)	
Hand	080 (9.0)	0080 (2.4)	
Others	010 (1.1)	0010 (0.3)	
Dominant vein			<0.01*
Cephalic	50 (56.2)	241 (72.4)	
Medial cubital	13 (14.6)	038 (11.4)	
Basilic	10 (11.2)	040 (12.0)	
Others	16 (18.0)	0140 (4.2)	
Successes at first catheterization attempts	56 (62.9)	239 (71.8)	<0.11
Unsuccessful	33 (37.1)	094 (28.2)	
Pharmacologic factor			
Hyperosmotic	33 (37.1)	041 (12.3)	<0.01*
Antibacterial	52 (58.4)	221 (66.4)	<0.20
Lipid solution	030 (3.4)	0030 (0.9)	<0.08

Data are presented as n (%) or mean ± SD. *p < 0.05.

Level of nurse experience: beginner, 0–100 PIVCs; intermediate, 101–800 PIVCs; expert, 801 + PIVCs.

BMI, body mass index; PIVC, peripheral intravenous catheter.

**Table 3:** Multiple logistic regression models for risk factors associated with catheter failure (n = 422).

	Model 1			Model 2			Model 3		
	OR	(95% CI)	*p* Value	OR	(95% CI)	*p* Value	OR	(95% CI)	*p* Value
Age, 0.1 year	1.00	(0.99–1.07)	0.63	1.00	(0.98–1.02)	0.97	1.001	(0.98–1.02)	0.97
Sex, male	0.63	(0.39–1.07)	0.09	0.73	(0.42–1.26)	0.25	0.682	(0.42–1.26)	0.25
BMI, 0.1	1.05	(0.98–1.02)	0.15	1.05	(0.98–1.12)	0.14	1.054	(0.98–1.12)	0.14
Care bundle intervention	0.30	(0.17–0.51)	0.01*	0.36	(0.20–0.64)	0.01*	0.357	(0.20–0.64)	0.01*
Hyperosmolar solutions				3.12	(1.74–5.57)	0.01*			
C-reactive protein, 0.1 mg/dL				1.06	(1.00–1.12)	0.04*	1.06	(1.00–1.12)	0.05*
Total un-used time (rocked time), 0.1 h				0.99	(0.99–1.00)	0.09	0.992	(0.99–1.00)	0.03*
Insertion anatomical site (compared with another site)				4.17	(1.79–9.70)	0.01*	4.081	(1.76–9.45)	0.01*
Number of catheterizations							1.462	(0.85–2.50)	0.17
Nagelkerke, R^2^			0.09			0.22			0.18

Hierarchical multiple logistic regression analysis was performed. Model 1 included age, sex, BMI, and care bundle intervention. Model 2 included Model 1 and hyperosmolar solutions, C-reactive protein, total un-used time, and insertion anatomical site (compared with another site). Model 3 included Model 1 and C-reactive protein, total unused time, and insertion anatomical site (compared with another site) and number of catheterizations.

OR: odds ratio. Model 3: Hosmer-Lemeshow test, *p* = 0.603.

### Confirmation of the effect of intervention

Table 4 shows the hierarchical multiple logistic regression analysis in the intervention group (n = 270). In model 4, entering “using polyurethane catheter” and “ultrasonography assist” showed significant difference regarding only “ultrasonography assist”, and this analysis showed that US assistance was more effective in preventing catheter failure than the use of polyurethane catheter in this study.

**Table 4:** Multiple logistic regression models for intervention items with catheter failure.

	Model 1			Model 2			Model 3			Model 4		
	OR	95% CI	*p* Value	OR	95% CI	*p* Value	OR	95% CI	*p* Value	OR	95% CI	*p* Value
Age (0.1 year)	1.01	(0.99–1.03)	0.47	1.00	(0.98–1.03)	0.55	1.00	(0.98–1.03)	0.69	1.01	(0.98–1.03)	0.70
Sex (Male)	0.74	(0.35–1.53)	0.41	0.73	(0.35–1.54)	0.41	0.70	(0.33–1.49)	0.36	0.70	(0.33–1.49)	0.36
BMI (0.1)	1.04	(0.96–1.13)	0.34	1.04	(0.96–1.13)	0.33	1.06	(0.97–1.15)	0.21	1.06	(0.97–1.15)	0.22
Hyperosmolar solutions	3.82	(1.65–8.83)	0.01*	3.78	(1.65–8.64)	0.01*	3.42	(1.48–7.91)	0.01*	3.39	(1.46–7.86)	0.01*
C-reactive protein (0.1 mg/dL)	1.03	(0.95–1.11)	0.54	1.01	(0.94–1.09)	0.76	0.99	(0.92–1.07)	0.88	0.99	(0.92–1.07)	0.87
Polyurethane catheter				0.60	(0.25–1.42)	0.24				0.88	(0.34–2.24)	0.78
Ultrasonography assisted							0.38	(0.18–0.80)	0.01*	0.39	(0.18–0.87)	0.02*
Nagelkerke R^2^			0.092			0.097			0.127			0.128

Hierarchical multiple logistic regression analysis was performed. Model 1 included age, sex, BMI, hyperosmolar solutions.

C-reactive protein. Model 2 included Model 1 and using polyurethane catheter. Model 3 included Model 1 and ultrasonography assist. Model 4 included all variables.

LL, lower limit; UL, upper limit; OR, odds ratio. Model 4: Hosmer-Lemeshow test, *p* = 0.19.

## Discussion

### New findings

To the best of our knowledge, this is the first study to prove that a care bundle for reducing mechanical irritation (including three intervention parameters, which were a pre-scan for assessment vein diameter, postscan for fixation according to assessment of catheter tip position in the vein, and using polyurethane catheter to prevent catheter failure) was effective. The findings supported the hypothesis confirming the effectiveness of the care bundle in catheter failure prevention based on vein diameter measurement and appropriate catheter tip position.

### Outcome interpretation

The incidence of catheter failure in the intervention and control groups was 11.1% and 29.2%, respectively. Compared to the control group, the intervention group had a relative risk reduction of >60% and NNT of 6.04, suggesting that the intervention was highly effective. Moreover, the care bundle prolonged catheter survival time by preventing catheter failure. This could be explained by mechanical irritation reduction because of selecting large diameter of vein and appropriate catheter tip position. Previous studies reported that mechanical irritation to vein wall by catheter was an important etiological factor for catheter failure^10,16,19,32^. This may cause local inflammation and coagulation and lead to thrombus and infection; to prevent these adverse effects along with mechanical irritation, several studies have reported development of catheter materials and fixation methods^24,33–39^. However, these studies could not evaluate the effectiveness for reducing catheter failure incidence because these trials did not observe actual veins and catheters. In the present study, using US to find the largest vein diameter (pre-scan) and the appropriate catheter tip position (post-scan) was effective in reducing mechanical irritation and solving the existing problem. Furthermore, because it was possible to identify the largest blood vessel, it was possible not only to achieve mechanical stimulation, but also potentially prevent thrombus formation due to those vessel’s enhanced blood flow.

Our previous study suggested that polyurethane catheter could reduce catheter failure^23^. This study might not show the effectiveness of the catheter material that was one intervention parameter compared with pre-scan for assessment vein diameter and post-scan for fixation according to the assessment of catheter tip position. Catheter failure caused by mechanical irritation might have been solved by selection of a larger-diameter vein and confirmation of the catheter position using US, which weakened the effect of the catheter material on catheter failure incidence. However, this analysis was conducted in a case where the care bundle was performed; therefore, it does not deny the effectiveness of the polyurethane catheter.

In the analysis for risk factors of catheter failure, hyperosmotic drugs, catheter placement on the back of the hands, and CRP were found to be associated. The catheter placement to hands has already been pointed out as a risk factor of catheter failure in many studies. The reason was that the insertion site was near the joint with wide range, the stability of the catheter is insufficient, and continuous mechanical stimulation might be placed on the vessel wall by the catheter. CRP was an indicator of the patient’s general inflammation condition and treatment situation and identifying its direct causal relationship is difficult.

The hyperosmotic solution such as amino acid solution was also a risk factor that has been pointed out^40–42^. Administration of high concentrations of drugs has been reported to result in development of severe ulcers^43,44^. This hyperosmotic solution was allowed to be administered via PIVC according to the guideline^45^. In this study, the use of hyperosmotic solution had three times higher risk for developing catheter failure. The odds ratio using this care bundle was one third and the use of hyperosmotic solution is almost equal in absolute effectiveness. The use of hyperosmotic solution almost eliminates the decrease in incidences of catheter failure on using the care bundles.

### Internal validity

There are three internal validity concerns, which are the research design, US examination method, and outcome evaluation challenges. First, regarding research design, this study was not an RCT. The effectiveness of the intervention was verified using a propensity score. When evaluating the propensity score, the fitness was good (C > 0.8), and the difference between the intervention and control groups in the almost feature quantities at baseline disappeared, i.e., that the propensity score matching was successful in making pseudo-randomized groups. In this study, the number of catheterizations catheterization times could not be completely adjusted via propensity score. However, independent patient analysis showed that this bias effect was minimal. There might be a risk for overestimation of catheter failure incidence, on using the Hawthorne effect^46^ because the nurses in the control group were aware that catheter failure was being evaluated due to non-blind study design. Therefore, the nurses in the control group might have been more careful to prevent catheter failure during the research period. However, in the control group, the incidence of catheter failure in this study was the same rate as in our previous study^7^. Therefore, this study showed that the care bundle was highly effective in reducing incidence of catheter failure.

Next, there was a threat of internal validity regarding the US examination method, and the quality of the intervention greatly differed. Generally, US operation depends on the operator’s skill and technique. Since this study was carried out by a researcher who was trained sufficiently, the quality of pre-scan and post-scan technique using US was well controlled.

The last concern was outcome evaluation challenges. In the present study, the assessment of catheter failure was performed by clinical nurses in two wards, which may have affected the incident rate of catheter failure depending on the nurse’s ability. However, there were no differences in the experience of catheter insertion times among nurses, and they received the same education using the hospital guideline for PIVC management in those two wards.

### External validity and clinical implementation

There were two external validity concerns, which were the target population and the intervention methods. The participants of this study were patients from urban areas who were hospitalized in an acute care university hospital and had an average age of about 65 years. There were many patients over 80 years who needed infusion therapy in long-term facilities. It may be difficult to detect large vessels in elderly people even through US; pre-scan and post-scan to detect the vessel and fixed catheter tip position were introduced.

External validity may be questioned in whether the nurses can perform US examination. Recently, some studies have reported the effectiveness of US examination in nursing care including the assessment of swallowing, urine volume, constipation, and pressure ulcers^47–54^. Convenient use of US like a stethoscope by a nurse could result in the provision of a higher quality of care^55^. When this care bundle for reducing catheter failure is implemented into actual clinical setting, the standardized education program of US examination to using PIVC for preventing catheter failure will be needed.

### Limitations

This study had two limitations. First, all US examinations were conducted by the researcher, and images were not blinded at the time of image acquisition. Second, the outcome was premature removal. In other words, even if the catheter was not removed, we could not find a patient with symptoms.

### Further research

High-concentration drug administration was found as a high-risk factor of catheter failure in this study. In cases of high-concentration drug administration, it is very popular to select central veins because mixing the drug with rich blood flow can reduce the chemical side effects to the local vein. One of the insertion catheters for the central vein is the peripherally inserted central catheter (PICC)^56–61^. However, a PICC cannot be used for all cases owing to the risk of mispuncture when approaching the central vein, severe infection, and thrombus^58,61,62^. Considering the home care setting in Japan for the future, it may be necessary to introduce new vascular access devices such as midline catheters instead of the PICC because the midline catheter can be placed in the basilic vein at the upper arm, which has rich blood flow, rather than a peripheral vein in the forearm to reduce the risk of infection and thrombus. Moreover, it is easier for nurses to use this type of catheter as a conventional PIVC. If we introduce a midline catheter place in the brachial vein, US guidance is needed. Healthcare providers including nurses need to learn US-guided insertion method. Therefore, it is necessary to establish a new safety system for midline catheterization with US guide. We plan to start developing a nursing education program and new vascular access device in collaboration with industries.

## Conclusion

This intervention study showed the significant reduction of catheter failure incidence using care bundle for reducing mechanical irritation, which included a pre-scan for the assessment vein diameter, post-scan for fixation according to the assessment of catheter tip position in the vein, and use of a polyurethane catheter in the clinical setting by nurses with the aid of US. This showed that the incidence of catheter failure in the intervention group was significantly lower than that in the control group.

## Methods

### Hypothesis

The care bundle reduces mechanical irritation and decreases the incidence of catheter failure compared to conventional care practices.

### Study design

An RCT design is considered ideal to examine intervention effects^63^. However, conducting an RCT is difficult owing to ethical concerns and feasibility in clinical setting. In this study, when examining the effect using RCT design in each catheter or patients, avoiding contamination among nurses working in the same department was difficult. Therefore, we adopted a non-randomized comparative non-blinding study design. This study was registered with the UMIN Clinical Trials Registry (UMIN000029850). The full trial protocol is available at https://upload.umin.ac.jp/cgi-open-bin/ctr/ctr_view.cgi?recptno = R000034094.

### Setting and recruitment

This study was conducted at the university of Toyo hospital in Japan between July and November 2017. The participants were recruited from two departments with high PIVC use, which was determined based on previous studies^64^, and no patients with extremely different attributes. Participants included patients ≥20 years who were hospitalized and received infusion therapy via a PIVC that was placed by nurses. Patients receiving chemotherapy and those with poor cognitive ability were excluded. The study procedures were explained to the doctors and nurses working in the ward at the beginning of the study period. Upon admission, patients who were expected to receive PIVC as part of their treatment were provided a written briefing of the study. Moreover, permission for patient intervention was obtained from the physician.

### Intervention procedures

The definition of intervention was performing three items in the care bundle as follows: pre-scan for assessment vein diameter, post-scan for fixation according assessment catheter tip position in the vein, and using a polyurethane catheter.

**Figure 3:**
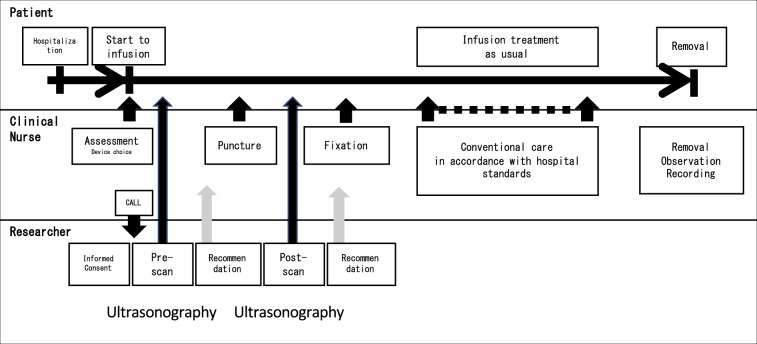
Diagram of using catheter flow in the intervention group.

**Figure 4:**
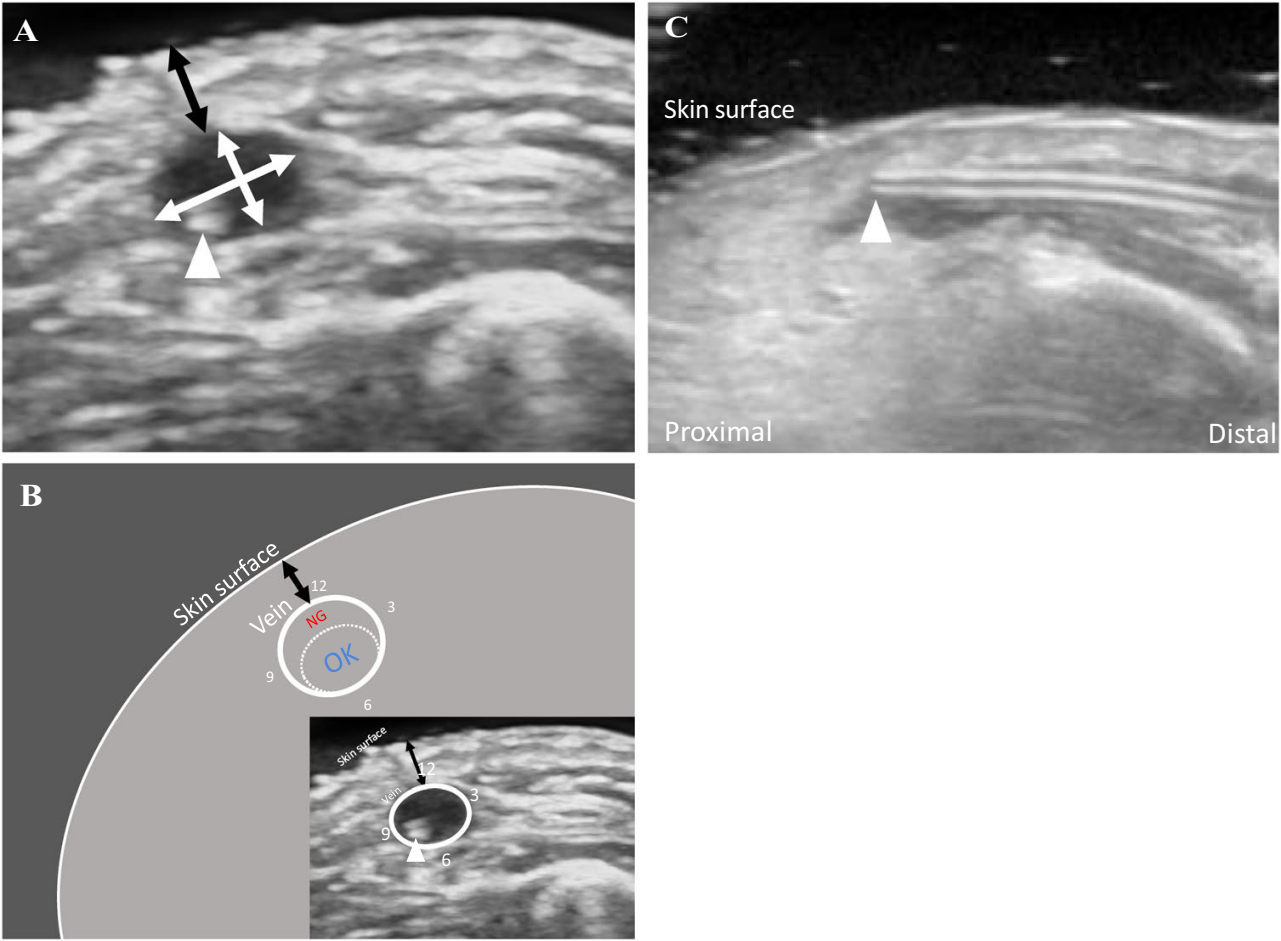
Definitions of measurement items in ultrasonographic images. (**A**) showed a short-axis ultrasonographic image of a vein with an inserted PIVC tip (arrow head). White arrows indicate the major and minor axis lengths. Vessel diameter was defined as [major axis + minor axis]/2. The major axis was the longest diameter. The minor axis was perpendicular to the major axis. Black arrows indicate depth defined as [distance from the skin surface to the nearest wall of vessel]. (**B**) showed schematic diagram of short axis image. Appropriate catheter tip position was defined as the location in the lower side or at the centre of the vein without being attached to the vessel wall (dashed circle). (**C**) showed a typical case that is no-good in post-scan. Normally, the catheter should lie along the blood vessel, but in this case it is lifted to the skin side. In such a state, mechanical stimulation is particularly enhanced. As a result, the catheter tip position is subject to adjustment. Abbreviation: PIVC, peripheral intravenous catheter.

Intervention procedures were performed as follows. Prior to the data collection period, nurses received lectures and underwent briefing sessions for 30 min. Figure 3 diagrams a timeline of catheter use in the intervention group. The procedure for the intervention group for each catheter was as follows. First, the nurse inserting the PIVC assessed the site to be punctured and relayed the findings to a researcher. Second, the researcher assessed vein size and location using US (pre-scan). Third, the researcher relayed the US examination results to the clinical nurse, who then decided the puncture point and performed the insertion. The nurses used a polyurethane catheter (Surflo^®^ V3; Terumo Corporation, Tokyo, Japan) in the intervention group. Depending on their comfort level, they chose either a Teflon or polyurethane catheter. After insertion, the researcher and clinical nurse observe and adjust the catheter tip position in the vein under US guidance (post-scan). The nurse carefully adjusted the catheter tip position to prevent pain due to unintentional removal of the catheter. We planned to complete the procedure within a maximum of one minute and stop immediately if the patient complains of discomfort or pain. Figure 4 shows the key ultrasound images and reference markers. The researcher measured vein diameter and depth and assessed catheter tip position using US as a surrogate outcome. Vein diameter was calculated as the sum of the major axis and minor axis divided by 2. The major axis was the longest diameter, and the minor axis was perpendicular to the major axis. The major and minor axes were measured three times using the US image (0.04 mm/pixel). The mean of the three measurements was used for analysis^22,65^. On identifying a target vein, the evaluators obtained a transverse image of the vein to measure the vein depth (distance from the skin surface to the superficial vein wall), and the mean of three measurements was used for analysis. The most appropriate catheter tip position was defined as the location in the lower side or centre of the vein that had no attached vein wall. Based on the post-scan, the tip was positioned within the blood vessel with careful attention to avoid pulling out the catheter. If contact deformed the blood vessel during insertion, it was immediately released. This was completed only in the cases where it was necessary.

The control group of patients was inserted with catheters using conventional care practices. The procedures for the control group were as follows: a nurse placed the PIVC and started intravenous therapy per routine practice with a Teflon^®^ catheter according to implementation criteria in the hospital. Within 24 hours after cannulation, a researcher visually inspected the condition of the PIVC puncture site (using a photographic device, visually inspecting the site, and asking the patient about their pain) and used an US to measure vein diameter and depth.

### Outcomes

The primary outcome was catheter failure (i.e., unplanned PIVC removal before completion of therapy), defined as a composite of the following catheter-related complications: complete dislodgement (the entire PIVC dislodged from the patient’s body), occlusion (the PIVC would not infuse or leakage was exhibited when fluid was infused), phlebitis (defined by the presence of one or more of the following symptoms: pain or tenderness, redness, swelling, and purulence with or without a palpable cord), or infection (presence of primary bloodstream infection or laboratory confirmed local PIVC infection), defined as unplanned removal of catheters based on standard nursing practice for using PIVC in this hospital, according to the Centers for Disease Control and Prevention guidelines. Information regarding catheter failure was obtained from the medical chart and interviews. Catheter insertion and removal, as well as data regarding time, were described in the medical charts at these wards. The researcher made direct observations at least twice a day, and interviewed nurses and patients as often as possible. The researcher also collected dwell time (survival rate) with the occurrence of problems with catheter failure.

### Other variables

The following data were collected by reviewing medical charts. Patient-related information included age, sex, body mass index (BMI), comorbidities (tumour or not at each organ), medical history (diabetes), and previous treatments (history of use of steroids, chemotherapeutic solutions, immunosuppressive solutions, anticoagulants, and radiation therapy); blood examination results included C-reactive protein (CRP), albumin, and platelet levels; administered medications at the baseline (use of hyperosmotic solutions, antibiotics, and fat emulsifiers); level of need for nursing (Kangodo [UMIN http://www.umin.ac.jp/kagoshima/]); and total time of locking. Nurse-related information included frequency of catheterization^66^. The following data were collected through macroscopic observation: characteristics of the catheterization site position (anatomical insertion site, dominant vein, success of first catheterization attempt, and number of catheterizations catheterization times) and PIVC type (catheter material and size). The characteristics of the target vein (diameter and depth) and catheter tip position in the vein were determined using US examination according to the methods described in a previous study^22^.

### Sample size and study power

The setting was as follows: main analysis method, comparison of proportions; two-sided chi-square test; sample size, 356 catheters (178 catheters per group) with an effect size of 0.15 (occurrence in the intervention group, 15%; occurrence in the control group, 30% according to previous study^7^), assuming a 10% dropout rate when setting α error = 0.05 and β error = 0.1 on power analysis. Data were collected over the same time period.

### Statistics

Statistical analysis was performed using SPSS version 22.0 (IBM Corp., New York, NY, USA) and JMP Pro software version 13.0.0 (SAS Institute, Cary, NC, USA). A *p* value < 0.05 was considered statistically significant.

The cumulative incidence rate of catheter failure during study periods, and the rate for each group per 1,000 device days as incidence rate (number of failures divided by the number of days catheterized then divided by 1,000) were calculated. We used Kaplan-Meier survival curves to compare rates of time until catheter failure between groups.

To minimize selection bias, we used inverse probability weighting (IPW)^67–69^. Propensity scores were calculated using multiple logistic regression to predict the probability of each patient receiving intervention, using all subject characteristic, including age, sex, BMI, primary disease, disease history, therapy (steroids, chemotherapy, immunosuppressant, radiation therapy, and anticoagulant), blood data (CRP, albumin, and platelet at baseline), number of catheterizations catheterization times, medicine administration, level of need for nursing (Kangodo), and level of nurse experience in PIVC insertion^66^. After propensity scores were calculated, intervention and control groups were balanced using IPW of propensity score.

Next, a multiple logistic regression analysis was used to estimate odds ratio (OR) and 95% confidence interval of factors contributing to catheter failure (n = 422). Variables with *p* values < 0.05 in the univariate analysis were selected to be entered into hierarchical logistic regression analysis. Spearman rank correlation coefficients among the candidates for analyses were calculated for continuous variables. If coefficients >0.4 were found between the independent variables, only one variable was entered in the model. If one variable for multiple logistic regression was a categorical variable, then the t-test or the chi-square test was used. If the *p* value was < 0.05, only one variable was entered. Age, sex, BMI, and performed intervention were selected as confounding factors.

Finally, subjects initially enrolled in the intervention group were included in the analysis (n = 270). Age, sex, BMI, anatomical site, and variables that were extracted from previous analysis were input as confounding variables, and the intervention contents added in this research, namely “catheter material” and “US assist,” were entered for.

### Ethics

The study protocol was approved by the Ethics Committee of the Graduate School of Medicine, The University of Tokyo (approval #10707). Written informed consent was obtained from all residents or their proxies. The study was performed in accordance with the principles of the Declaration of Helsinki.

### Consent for publication

All authors approved the final manuscript in the submitted version and declare themselves in agreement that they agree to all aspects of the work and consent to publication.

## Supplementary information

Supplements tables.

Study protocol.

## Data Availability

This study was registered with the UMIN Clinical Trials Registry (UMIN000029850). Date of protocol fixation: 01 July 2017. Anticipated trial start date: 01 July 2017. The full date of registration: 06 Nov 2017. The full trial protocol is available at https://upload.umin.ac.jp/cgi-open-bin/ctr_e/ctr_view.cgi?recptno=R000034094.

## References

[1] Waitt. C, Waitt. P, Pirmohamed M (2003). Intravenous therapy. Postgrad. Med. J..

[2] Ritchie S, Jowitt D, Roberts S (2007). & Auckland District Health Board Infection Control, S. The Auckland City Hospital Device Point Prevalence Survey 2005: utilisation and infectious complications of intravascular and urinary devices. N. Z. Med. J..

[3] Pujol M (2007). Clinical epidemiology and outcomes of peripheral venous catheter-related bloodstream infections at a university-affiliated hospital. J. Hosp. Infect..

[4] Koh DB, Gowardman JR, Rickard CM, Robertson IK, Brown A (2008). Prospective study of peripheral arterial catheter infection and comparison with concurrently sited central venous catheters. Crit. Care Med..

[5] Rickard CM (2012). Routine versus clinically indicated replacement of peripheral intravenous catheters: a randomised controlled equivalence trial. Lancet.

[6] Murayama, R. *et al*. Patient risk factors for developing sign- and symptom-related peripheral intravenous catheter failure: A retrospective study. *J. Japanese Soc. Wound, Ostomy, Cont. Manag.* **19**, 13594–13402 (2015).

[7] Takahashi T (2017). Is Thrombus With Subcutaneous Edema Detected by Ultrasonography Related to Peripheral Intravenous Catheter Failure?. J. Infus. Nurs..

[8] Wallis MC (2014). Risk factors for peripheral intravenous catheter failure: a multivariate analysis of data from a randomized controlled trial. Infect. Control. Hosp. Epidemiol..

[9] Limm EI, Fang X, Dendle C, Stuart RL, Egerton Warburton D (2013). Half of all peripheral intravenous lines in an Australian tertiary emergency department are unused: pain with no gain?. Ann. Emerg. Med..

[10] Zingg W, Pittet D (2009). Peripheral venous catheters: an under-evaluated problem. Int. J. Antimicrob. Agents.

[11] Hadaway L (2012). Short peripheral intravenous catheters and infections. J. Infus. Nurs..

[12] Webster J (2008). Routine care of peripheral intravenous catheters versus clinically indicated replacement: randomised controlled trial. BMJ.

[13] Dychter SS, Gold DA, Carson D, Haller M (2012). Intravenous therapy: a review of complications and economic considerations of peripheral access. J. Infus. Nurs..

[14] Tagalakis V, Kahn SR, Libman M, Blostein M (2002). The epidemiology of peripheral vein infusion thrombophlebitis: a critical review. Am. J. Med..

[15] Everitt NJ, Krupowicz DW, Evans JA, McMahon MJ (1997). Ultrasonographic investigation of the pathogenesis of infusion thrombophlebitis. Br. J. Surg..

[16] Weiss D, Yaakobovitch H, Tal S, Nyska A, Rotman OM (2019). Novel short peripheral catheter design for prevention of thrombophlebitis. J. Thromb. Haemost..

[17] Weiss D, Gefen A, Einav S (2016). Modelling catheter-vein biomechanical interactions during an intravenous procedure. Comput. Methods Biomech. Biomed. Engin.

[18] Weiss D (2017). Mechanical Compression Effects on the Secretion of vWF and IL-8 by Cultured Human Vein Endothelium. PLoS One.

[19] Weiss D, Rotman OM, Einav S (2017). Quantitative T2 mapping for detection and quantification of thrombophlebitis in a rabbit model. J. Biomech..

[20] Murayama R (2015). The relationship between the tip position of an indwelling venous catheter and the subcutaneous edema. Biosci. Trends.

[21] Takahashi T (2018). Observational study at the time of peripheral venous catheter placement using the ultrasonography and qualitative sketching method. J. Nurs. Sci. Engineering..

[22] Tanabe H (2016). Using ultrasonography for vessel diameter assessment to prevent infiltration. J. Infus. Nurs..

[23] Tanabe H (2016). Low-angled peripheral intravenous catheter tip placement decreases phlebitis. J. Vasc. Access..

[24] Maki DG, Ringer M (1991). Risk factors for infusion-related phlebitis with small peripheral venous catheters. A randomized controlled trial. Ann. Intern. Med..

[25] Stanley MD, Meister E, Fuschuber K (1992). Infiltration during intravenous therapy in neonates: comparison of Teflon and Vialon catheters. South. Med. J..

[26] Pinto TJ, Ribeiro AD (1999). The influence of the intravenous catheter composition on its hemocompatibility. PDA J. Pharm. Sci. Technol..

[27] Takashima M, R.-B. G., Keogh S, Rickard CM (2015). Randomised controlled trials in peripheral vascular access catheters: a scoping review. Vasc. Access..

[28] Resar R (2005). Using a bundle approach to improve ventilator care processes and reduce ventilator-associated pneumonia. Jt. Comm. J. Qual. Patient Saf..

[29] Burger CD, Resar RK (2006). “Ventilator bundle” approach to prevention of ventilator-associated pneumonia. Mayo Clin. Proc..

[30] Pan Y (2017). Microbial investigations in throat swab and tracheal aspirate specimens are beneficial to predict the corresponding endotracheal tube biofilm flora among intubated neonates with ventilator-associated pneumonia. Exp. Ther. Med..

[31] Mestre G (2013). Successful multifaceted intervention aimed to reduce short peripheral venous catheter-related adverse events: a quasiexperimental cohort study. Am. J. Infect. Control..

[32] Rotman OM, Shav D, Raz S, Zaretsky U, Einav S (2013). Biomechanical aspects of catheter-related thrombophlebitis. J. Biomed. Sci. Eng..

[33] Maki DG, Ringer M (1987). Evaluation of dressing regimens for prevention of infection with peripheral intravenous catheters. Gauze, a transparent polyurethane dressing, and an iodophor-transparent dressing. JAMA.

[34] Soriano E (2015). Fixing device for closing and coupling an intravenous catheter. Nutr. Hosp..

[35] Naimer SA, Temira F (2004). Evaluation of techniques for intravenous catheter and tubing fixation. Mil. Med..

[36] Lundgren A, Jorfeldt L, Ek AC (1993). The care and handling of peripheral intravenous cannulae on 60 surgery and internal medicine patients: an observation study. J. Adv. Nurs..

[37] Madeo M, Martin C, Nobbs A (1997). A randomized study comparing IV 3000 (transparent polyurethane dressing) to a dry gauze dressing for peripheral intravenous catheter sites. J. Intraven. Nurs..

[38] Takashima M, Ray-Barruel G, Ullman A, Keogh S, Rickard CM (2017). Randomized controlled trials in central vascular access devices: A scoping review. PLoS One.

[39] Takahashi T (2019). Catheter tips are a possible resource for biological study on catheter failure. Drug. Discoveries Therapeutics.

[40] Everitt NJ (1999). Effect of prolonged infusion on vein calibre: a prospective study. Ann. R. Coll. Surg. Engl..

[41] Monreal M (1999). Infusion phlebitis in patients with acute pneumonia: a prospective study. Chest.

[42] Brandt CT (2000). Phlebitis due to venous catheters. Causes and occurrence. Ugeskr. Laeger.

[43] Roberts GW (1994). Peripheral intravenous line survival and phlebitis prevention in patients receiving intravenous antibiotics: heparin/hydrocortisone versus in-line filters. Ann. Pharmacother..

[44] Myrianthefs P, Sifaki M, Samara I, Baltopoulos G (2005). The epidemiology of peripheral vein complications: evaluation of the efficiency of differing methods for the maintenance of catheter patency and thrombophlebitis prevention. J. Eval. Clin. Pract..

[45] Boullata JI (2014). A.S.P.E.N. clinical guidelines: parenteral nutrition ordering, order review, compounding, labeling, and dispensing. JPEN J. Parenter. Enter. Nutr..

[46] Eckmanns T, Bessert J, Behnke M, Gastmeier P, Ruden H (2006). Compliance with antiseptic hand rub use in intensive care units: the Hawthorne effect. Infect. Control. Hosp. Epidemiol..

[47] Miura Y (2016). Detecting pharyngeal post-swallow residue by ultrasound examination: a case series. Med. Ultrason..

[48] Miura Y (2014). Method for detection of aspiration based on B-mode video ultrasonography. Radiol. Phys. Technol..

[49] Yabunaka K (2009). Can ultrasonographic evaluation of subcutaneous fat predict pressure ulceration?. J. Wound Care.

[50] Yabunaka K, Nakagami G, Komagata K, Sanada H (2017). Ultrasonographic follow-up of functional chronic constipation in adults: A report of two cases. SAGE Open. Med. Case Rep..

[51] Yabunaka K (2011). Sonographic assessment of hyoid bone movement during swallowing: a study of normal adults with advancing age. Radiol. Phys. Technol..

[52] Yabunaka K, Iizaka S, Nakagami G, Fujioka M, Sanada H (2015). Three-dimensional ultrasound imaging of the pressure ulcer. A case report. Med. Ultrason..

[53] Aoi N (2009). Ultrasound assessment of deep tissue injury in pressure ulcers: possible prediction of pressure ulcer progression. Plast. Reconstr. Surg..

[54] Nagase T (2007). Ultrasonographic evaluation of an unusual peri-anal induration: a possible case of deep tissue injury. JWC.

[55] Christopher L, Moore MD, Joshua A, Copel MD (2011). Point-of-Care Ultrasonography. N. Engl. J. Med..

[56] Chopra V (2017). Vascular Access Specialist Training, Experience, and Practice in the United States: Results From the National PICC1 Survey. J. Infus. Nurs..

[57] Rickard CM (2017). Peripherally inserted central catheter dressing and securement in patients with cancer: the PISCES trial. Protocol for a 2x2 factorial, superiority randomised controlled trial. BMJ Open..

[58] Chopra V, Kuhn L, Ratz D, Flanders SA, Krein SL (2016). Vascular nursing experience, practice knowledge, and beliefs: Results from the Michigan PICC1 survey. J. Hosp. Med..

[59] Chopra V (2015). The Michigan Appropriateness Guide for Intravenous Catheters (MAGIC): Results from a multispecialty panel using the RAND/UCLA appropriateness method. Ann. Intern. Med..

[60] Moureau N, Chopra V (2016). Indications for peripheral, midline and central catheters: summary of the MAGIC recommendations. Br. J. Nurs..

[61] Woller SC, Stevens SM, Evans RS (2016). The Michigan Appropriateness Guide for Intravenous Catheters (MAGIC) initiative: A summary and review of peripherally inserted central catheter and venous catheter appropriate use. J. Hosp. Med..

[62] Chopra V (2013). Risk of venous thromboembolism associated with peripherally inserted central catheters: a systematic review and meta-analysis. Lancet.

[63] Rossi, P. H., Lipsey, M. & Freeman H. E. Evaluation: a systematic approach. *Thousand Oaks, CA: Sage* (2004).

[64] Murayama R (2017). Removal of peripheral intravenous catheters due to catheter failures among adult patients. J. Infus. Nurs..

[65] Takahashi Toshiaki, Murayama Ryoko, Yabunaka Koichi, Tanabe Hidenori, Sanada Hiromi (2019). Using Tablet-Type Ultrasonography to Assess Peripheral Veins for Intravenous Catheterization: A Pilot Study. Journal of the Association for Vascular Access.

[66] Rippey JC, Carr PJ, Cooke M, Higgins N, Rickard CM (2016). Predicting and preventing peripheral intravenous cannula insertion failure in the emergency department: Clinician ‘gestalt’ wins again. Emerg. Med. Australas..

[67] Paul R (1983). Rosenbaum & Rubin, D. B. The central role of the propensity score in observational studies for causal effects. Biometrika.

[68] Hoshino T, Okada K (2006). Estimation of Causal Effect Using Propensity Score Methods in Clinical Medicine, Epidemiology, Pharmacoepidemiology and Public Health; A Review. J. Natl. Inst. Public. Health.

[69] Linden A, Adams JL (2010). Using propensity score-based weighting in the evaluation of health management programme effectiveness. J. Eval. Clin. Pract..

