# Supplementary material for: Preventing peripheral intravenous catheter failure by reducing mechanical irritation
Source: Sci Rep. 2020 Jan 31;10:1550. doi: 10.1038/s41598-019-56873-2 (PMC6994694; doi:10.1038/s41598-019-56873-2)
Supplement: Supplementary file 1 — Supplements tables. [file 41598_2019_56873_MOESM1_ESM.docx]

Preventing peripheral intravenous catheter failure by reducing mechanical irritation

Toshiaki Takahashi; Ryoko Murayama; Mari Abe; Maki Miyahara; Chiho Kanno; Miwa Nakamura; Mariko Mizuno; Chieko Komiyama; Hiromi Sanada

Toshiaki Takahashi^1^, PhD, RN;

Ryoko Murayama^2,3^, PhD, RN, RMW;

Mari Abe^2^, MHS, RN;

Maki Miyahara^4^, MHS, RN;

Chiho Kanno^4^, RN;

Miwa Nakamura^5^, RN;

Mariko Mizuno^5^, RN;

Chieko Komiyama^5^, MNS, RN;

Hiromi Sanada*^3,4^, PhD, RN, WOCN

^1^Department of Life Support Technology (Molten) Graduate School of Medicine, the University of Tokyo

^2^Department of Advanced Nursing Technology, Graduate School of Medicine, The University of Tokyo

^3^Global Nursing Research Center, Graduate School of Medicine, The University of Tokyo

^4^Department of Gerontological Nursing/Wound Care Management, Graduate School of Medicine, The University of Tokyo

^5^Department of Nursing, The University of Tokyo Hospital, Japan.

Toshiaki Takahashi Email: takahashito-tky@umin.ac.jp

Ryoko Murayama Email: rymurayama-tky@umin.ac.jp

Mari Abe Email: mdoi-tky@umin.ac.jp

Maki Miyahara Email: komaki-tky@umin.ac.jp

Chiho Kanno Email: ckanno-tky@umin.ac.jp

Miwa Nakamura Email: NAKAMURAM-NUR@h.u-tokyo.ac.jp

Mariko Mizuno Email: MIZUNOM-NUR@h.u-tokyo.ac.jp

Chieko Komiyama Email: komiyamac@adm.h.u-tokyo.ac.jp

Hiromi Sanada E-mail: hsanada-tky@umin.ac.jp

Corresponding author:

Hiromi Sanada,

Department of Gerontological Nursing/Wound Care Management, Graduate School of Medicine, The University of Tokyo, Faculty of Medicine Bldg. No. 5-306, 7-3-1, Hongo, Bunkyo-ku, Tokyo 113-0033, Japan
E-mail: hsanada-tky@umin.ac.jp

Tel.: +81-3-5841-3442
Fax: +81-3-5841-3442

Supplements

| Table S1 Characteristics of catheter and patient in each group | | |  |  |  |  |  |  |  |  |  |  |  |  |
| --- | --- | --- | --- | --- | --- | --- | --- | --- | --- | --- | --- | --- | --- | --- |
|  |  | Control group | | | | | |  | Intervention group | | | | | |
|  |  | Per catheter | | Per patient | | Drop out | |  | Per catheter | | Per patient | | Drop out | |
|  |  | 233 |  | 157 |  | 79 |  |  | 189 |  | 160 |  | 81 |  |
| Gender | |  |  |  |  |  |  |  |  |  |  |  |  |  |
|  | Male | 162 | (69.5) | 110 | (70.1) | 53 | (67.1) |  | 124 | (65.6) | 106 | (66.3) | 57 | (70.4) |
| Age; year | | 66.4 | ±15.9 | 66.8 | ±14.2 | 67.4 | ±14.2 |  | 65.2 | ±14.3 | 65.8 | ±14.1 | 70.7 | ±14.9 |
| BMI | | 23.1 | ±3.4 | 23.1 | ±3.4 | 22.8 | ±3.0 |  | 22.9 | ±4.1 | 23.2 | ±4.0 | 21.8 | ±3.6 |
| Present illness (tumor) | |  |  |  |  |  |  |  |  |  |  |  |  |  |
|  | Tumor | 96 | (41.2) | 72 | (45.9) | 28 | (35.4) |  | 121 | (64.0) | 107 | (66.9) | 42 | (51.9) |
| Present illness (each organ) | |  |  |  |  |  |  |  |  |  |  |  |  |  |
|  | Gastroenterology | 62 | (26.6) | 44 | (28.0) | 21 | (26.6) |  | 50 | (26.5) | 45 | (28.1) | 25 | (31.6) |
|  | Hepatobiliary Pancreatic | 153 | (65.7) | 99 | (63.1) | 41 | (51.9) |  | 129 | (68.3) | 108 | (67.5) | 45 | (57.0) |
|  | Other | 18 | (7.7) | 14 | (8.9) | 17 | (21.5) |  | 10 | (5.3) | 7 | (4.4) | 11 | (13.9) |
| Diabetes | | 39 | (16.7) | 30 | (19.1) | 16 | (20.3) |  | 44 | (23.3) | 38 | (23.8) | 20 | (24.7) |
| Oral medicine (anticoagulant) | | 35 | (15.0) | 28 | (17.8) | 14 | (17.7) |  | 26 | (13.8) | 23 | (14.4) | 11 | (13.6) |
| Oral medicine (steroid) | | 8 | (3.4) | 8 | (5.1) | 1 | (1.3) |  | 12 | (6.3) | 11 | (6.9) | 2 | (2.5) |
| Chemotherapy | | 10 | (4.3) | 5 | (3.2) | 3 | (3.8) |  | 10 | (5.3) | 7 | (4.4) | 5 | (6.2) |
| Radiation | | 1 | (0.4) | 1 | (0.6) | 1 | (1.3) |  | 1 | (0.5) | 1 | (0.6) | 4 | (4.9) |
| The level of need for nursing (KANGODO) | |  |  |  |  |  |  |  |  |  |  |  |  |  |
|  | A1 | 1 | (0.4) | 1 | (0.6) | 3 | (3.8) |  | 2 | (1.1) | 2 | (1.3) | 2 | (2.5) |
|  | A2 | 4 | (1.7) | 3 | (1.9) | 2 | (2.5) |  | 2 | (1.1) | 1 | (0.6) | 6 | (7.4) |
|  | A3 | 4 | (1.7) | 3 | (1.9) | 4 | (5.1) |  | 2 | (1.1) | 1 | (0.6) | 3 | (3.7) |
|  | A4 | 1 | (0.4) | 1 | (0.6) | 0 | (0.0) |  | 0 | (0.0) | 0 | (0.0) | 0 | (0.0) |
|  | B1 | 19 | (8.2) | 14 | (8.9) | 14 | (17.7) |  | 13 | (6.9) | 9 | (5.6) | 6 | (7.4) |
|  | B2 | 5 | (2.1) | 5 | (3.2) | 3 | (3.8) |  | 8 | (4.2) | 6 | (3.8) | 8 | (9.9) |
|  | B3 | 108 | (46.4) | 58 | (36.9) | 33 | (41.8) |  | 70 | (37.0) | 57 | (35.6) | 27 | (33.3) |
|  | B4 | 51 | (21.9) | 38 | (24.2) | 11 | (13.9) |  | 38 | (20.1) | 33 | (20.6) | 14 | (17.3) |
|  | C1 | 0 | (0.0) | 0 | (0.0) | 0 | (0.0) |  | 0 | (0.0) | 0 | (0.0) | 0 | (0.0) |
|  | C2 | 0 | (0.0) | 0 | (0.0) | 0 | (0.0) |  | 0 | (0.0) | 0 | (0.0) | 0 | (0.0) |
|  | C3 | 2 | (0.9) | 2 | (1.3) | 1 | (1.3) |  | 5 | (2.6) | 5 | (3.1) | 1 | (1.2) |
|  | C4 | 38 | (16.3) | 32 | (20.4) | 8 | (10.1) |  | 49 | (25.9) | 46 | (28.8) | 13 | (16.0) |
| Blood examination | |  |  |  |  |  |  |  |  |  |  |  |  |  |
|  | C-reactive protein | 2.81 | ±4.7 | 2.1 | ±14.2 | 3.8 | ±5.6 |  | 1.08 | ±2.5 | 1.0 | ±2.5 | 3.7 | ±4.2 |
|  | Albumin | 3.5 | ±0.7 | 3.8 | ±0.7 | 3.3 | ±0.9 |  | 3.8 | ±0.7 | 3.8 | ±0.7 | 3.4 | ±0.9 |
|  | Platelet | 21.8 | ±10.9 | 20.7 | ±10.7 | 21.4 | ±12.0 |  | 20.4 | ±9.2 | 19.9 | ±9.2 | 21.7 | ±10.1 |
| No. of entry | |  |  |  |  |  |  |  |  |  |  |  |  |  |
|  | Once |  |  | 107 | (68.2) |  |  |  |  |  | 139 | (86.9) |  |  |
|  | Twice |  |  | 33 | (21.0) |  |  |  |  |  | 20 | (12.5) |  |  |
|  | Three times or more |  |  | 15 | (9.6) |  |  |  |  |  | 2 | (1.3) |  |  |
| Notes: n (%) ±SD, Abbreviations: PIVC, peripheral intravenous catheter; | | | | | | | | | | | | | | |

| Table S2. Incidence of catheter failure **per patient** | | |
| --- | --- | --- |
|  | No. of catheter failure incidence (%) | *P* value |
| Intervention  (n=160) | 16 (10.0) | < 0.001 |
| Control (n=157) | 43 (27.4) |  |
| Analysis by Chi-squared test | | |

| Table S3. Incidence of catheter failure **per patient** weighted by inverse probability of propensity score | | | |
| --- | --- | --- | --- |
|  | Rate of catheter failure incidence (%) | RRR (95% CI) | NNT (95% CI) |
| Intervention | 11.0 | 0.63 (0.34 to 0.80) | 7.35 (5.08 to 16.95) |
| Control | 27.6 |  |  |
| RRR, Relative risk reduction; CI, confidence interval; NNT, number needed to treat;  RRR was calculated as Rate of catheter failure incidence in control - Rate of catheter failure incidence in intervention) divided rate of catheter failure incidence in control  NNT was calculated 1/(Rate of catheter failure incidence in control - Rate of catheter failure incidence in intervention) | | | |
