# Supplementary material for: Preventing peripheral intravenous catheter failure by reducing mechanical irritation
Source: Sci Rep. 2020 Jan 31;10:1550. doi: 10.1038/s41598-019-56873-2 (PMC6994694; doi:10.1038/s41598-019-56873-2)
Supplement: Supplementary file 2 — Study protocol. [file 41598_2019_56873_MOESM2_ESM.pdf]

Recommended format for a Research Protocol

Part 1

Project summary

Peripheral intravenous catheter failure is a significant concern in the clinical setting.
We investigated the effectiveness of care protocols, including an ultrasonographic "prescan" for selecting a large-diameter vein before catheterization, a "postscan" for confirming the catheter tip position after catheterization with ultrasonography, and the use of a flexible polyurethane catheter to reduce the mechanical irritation that contributes to the incidence of catheter failure.
This intervention study was a non-randomized controlled trial to investigate the effectiveness of the abovementioned care protocols, the effects of which were compared to the outcomes in the control group, which received conventional care. All participants were selected from patients in two wards (intervention and control) at the University of Tokyo in Japan, between July and November 2017. Inverse probability score-based weighted methods (IPW) using propensity score were used to estimate the effectiveness of care protocols. The primary outcome was catheter failure, which was defined as accidental and unplanned catheter removal. We used Kaplan-Meier survival curves to compare rates of time until catheter failure.
We analysed 189 and 233 catheters in the intervention and control groups, respectively. In the control group, 68 catheters (29.2%) were determined to have failed, whereas in the intervention group, only 21 catheters (11.1%) failed. There was a significant difference between each group in terms of the ratio of catheter failure adjusted according to IPW ($p = 0.003$). The relative risk reduction of the intervention for catheter failure was 0.60 (95% CI: 0.47-0.71).
Care protocols, including assessment of vein diameter, vein depth, and catheter tip location using ultrasound examination for reducing mechanical irritation is a promising method to reduce catheter failure incidence.

General information

This study was registered with the UMIN Clinical Trials Registry (UMIN000029850). The full trial protocol is available at https://upload.umin.ac.jp/cgi-open-bin/ctr/ctr_view.cgi?recptno=R000034094.

Anticipated trial start date: 01 July 2017

Corresponding author: Hiromi Sanada,
Department of Gerontological Nursing/Wound Care Management, Graduate School of Medicine, The University of Tokyo, Faculty of Medicine Bldg. No. 5-306, 7-3-1, Hongo, Bunkyo-ku, Tokyo 113-0033, Japan
E-mail: hsanada-tky@umin.ac.jp
Tel.: +81-3-5841-3442 Fax: +81-3-5841-3442

Funding
This work was supported by a JSPS grant (No. 16H05561) and Yamaji Fumiko Nursing
Japan Society for the Promotion of Science (JSPS)
5-3-1 Kojimachi, Chiyoda-ku, Tokyo 102-0083, JAPAN
Yamaji Fumiko Nursing.
3-33-1 Shiba, Minato-ku, Tokyo 105-8574, JAPAN

Rationale & background information

Catheter failure

Most patients require at least one peripheral vascular device for delivering intravenous fluids and medications during their hospital stay. A peripheral intravenous catheter (PIVC) is commonly used. Recent studies reported that >70% of patients in acute care hospitals use PIVCs (1-3). Additionally, >25% of PIVCs are reportedly accidentally removed, termed catheter failure (3-5). In a study conducted over 2 months at a university hospital in Tokyo, Japan, 5,316 catheters from 2,442 patients were studied; the rate of catheter removal due to catheter failure was 18.8%(6). Catheter failure is associated with signs and symptoms such as erythema, swelling, induration, bleeding, pain, and insufficient dripping (5, 7, 8). These symptoms negatively affect patient comfort and treatment, eventually making it difficult to continue intravenous therapy (1, 9). In such cases, catheters need to be replaced, which makes patients uncomfortable, increases labour, and drives up costs

(10-12). Therefore, it is important for both patients and healthcare providers to prevent catheter failure in PIVC.

Preventing catheter failure

Risk factors of various PIVC complications are known, but healthcare providers cannot prevent them (e.g., phlebitis, infiltration) sufficiently (13-15) (13, 16, 17). Although equipment, technology, and education improve daily, these problems still exist, causing patients to feel uncomfortable and discontinue treatment. The current preventive measures are insufficient; novel measures are needed. Our previous study suggested that mechanical irritation was an important factor in catheter failure (7). Therefore, we focused on mechanical irritation. Our laboratory reported that PIVC tip contact with the vessel wall was associated with subcutaneous oedema (18). The patient's posture during insertion and fixation could affect thrombus and oedema formation, as mechanical irritation affects the veins and subcutaneous tissue.

Next, we analysed the relationship between "vein diameter and catheter failure". Our laboratory analysed vein diameter and calculated the ratios of the vein diameter to the catheter gauge under the same conditions as those in the previous research (19). The relationship between these ratios and infiltration was assessed to determine a cut-off point. The mean ratio of vein diameter in the infiltration group was significantly smaller than in the no-infiltration group ($p < 0.01$), and the ratio was an independent risk factor according to the multivariable analysis. The ratio of 3.3 times was determined to be the cut-off point that enables healthcare providers to identify veins appropriately. For example, if a 22-gauge catheter is selected, a site where vein diameter exceeds 3.0 mm is suitable.

Last, the relationship of the catheter material, angle, and failure was analysed using ultrasonography (US) (20). This study suggested that a polyurethane catheter was effective in preventing catheter failure due to its softness. Our previous study on the risk factors for phlebitis reported an association between the use of polyurethane catheters and a 30%-50% reduction in phlebitis occurrence compared to after using polytetrafluorethylene (Teflon®) catheters (21-23). In this study, therefore, we also included the use of a polyurethane catheter.

Therefore, our laboratory focused on "damage to the vein by mechanical irritation" including three points, which were "appropriate catheter tip position", "vein diameter", and "catheter material".

Care bundle approach for preventing catheter failure

Despite numerous randomized controlled trials (RCTs) on PIVC worldwide (24) catheter

failure remains unresolved. This may be because intravenous therapy involves multiple steps, including site selection, catheterization, and fixation; performing one or some of these processes well is insufficient. Rather than focusing on the outcomes of each process, it is essential to introduce the outcome of all processes simultaneously. The approach for such interventions is akin to the care bundle approach, which is employed in clinical settings for controlling infection and preventing ventilator-associated pneumonia (25-27). A care bundle is defined as a "set of processes, generally three to five, that are proven by RCTs and are performed as a whole to obtain optimal outcomes" (25). Using a bundle approach to develop care practices for controlling infection and preventing ventilator-associated pneumonia yields positive clinical outcomes (26). In PIVC infection management, performing multiple intervention has been shown to yield certain positive outcomes (28), but the care bundle approach has not been reported to prevent catheter failure. In our laboratory, several studies have yielded implications for clinical care. These studies provide evidence for processes such as appropriate selection of puncture site and device and fixation method. We believe that conducting the intervention using bundled concepts is possible, especially when focused on preventing catheter failure by reducing mechanical irritation. In this study, we bundled three points extracted from the analysis of our previous research and called it a "care bundle", that is, "prescan" for selecting a large vein diameter before catheterization, "postscan" for confirming the catheter tip position after catheterization, and "use of polyurethane catheter" with more flexibility.

References (of literature cited in preceding sections)

1. Waitt. C, Waitt. P, Pirmohamed M. Intravenous therapy. Postgrad Med J. 2003;80:1-6.
2. Ritchie S, Jowitt D, Roberts S, Auckland District Health Board Infection Control S. The Auckland City Hospital Device Point Prevalence Survey 2005: utilisation and infectious complications of intravascular and urinary devices. N Z Med J. 2007;120:U2683.
3. Pujol M, Hornero A, Saballs M, Argerich MJ, Verdaguer R, Cisnal M, et al. Clinical epidemiology and outcomes of peripheral venous catheter-related bloodstream infections at a university-affiliated hospital. J Hosp Infect. 2007;67:22-9.
4. Koh DB, Gowardman JR, Rickard CM, Robertson IK, Brown A. Prospective study of peripheral arterial catheter infection and comparison with concurrently sited central venous catheters. Crit Care Med. 2008;36:397-402.
5. Rickard CM, Webster J, Wallis MC, Marsh N, McGrail MR, French V, et al. Routine

versus clinically indicated replacement of peripheral intravenous catheters: a randomised controlled equivalence trial. Lancet. 2012;380:1066-74.
6. Murayama. R, Uchida. M, Oe. M, Takahashi. T, Oya. M, Komiyama. C, et al. Patient risk factors for developing sign- and symptom-related peripheral intravenous catheter failure: A retrospective study. J Jpn WOCM. 2015;19:13594-402.
7. Takahashi T, Murayama R, Oe M, Nakagami G, Tanabe H, Yabunaka K, et al. Is thrombus with subcutaneous edema detected by ultrasonography related to short peripheral catheter failure? a prospective observational study. J Infus Nurs. 2017;40:313-22.
8. Wallis MC, McGrail M, Webster J, Marsh N, Gowardman J, Playford EG, et al. Risk factors for peripheral intravenous catheter failure: a multivariate analysis of data from a randomized controlled trial. Infect Control Hosp Epidemiol. 2014;35:63-8.
9. Limm EI, Fang X, Dendle C, Stuart RL, Egerton Warburton D. Half of all peripheral intravenous lines in an Australian tertiary emergency department are unused: pain with no gain? Ann Emerg Med. 2013;62:521-5.
10. Zingg W, Pittet D. Peripheral venous catheters: an under-evaluated problem. Int J Antimicrob Agents. 2009;34 Suppl 4:S38-42.
11. Hadaway L. Short peripheral intravenous catheters and infections. J Infus Nurs. 2012;35:230-40.
12. Webster J, Clarke S, Paterson D, Hutton A, van Dyk S, Gale C, et al. Routine care of peripheral intravenous catheters versus clinically indicated replacement: randomised controlled trial. BMJ. 2008;337:a339.
13. Dychter SS, Gold DA, Carson D, Haller M. Intravenous therapy: a review of complications and economic considerations of peripheral access. J Infus Nurs. 2012;35:84-91.
14. Tagalakis V, Kahn SR, Libman M, Blostein M. The epidemiology of peripheral vein infusion thrombophlebitis: a critical review. Am J Med. 2002;113:146-51.
15. Everitt NJ, Krupowicz DW, Evans JA, McMahon MJ. Ultrasonographic investigation of the pathogenesis of infusion thrombophlebitis. Br J Surg. 1997;84:642-5.
16. Dougherty L. IV therapy: recognizing the differences between infiltration and extravasation. Br J Nurs. 2008;17:896-1.
17. Marsh N, Webster J, Larson E, Cooke M, Mihala G, Rickard CM. Observational study of peripheral intravenous catheter outcomes in adult hospitalized patients: a multivariable analysis of peripheral intravenous catheter failure. J Hosp Med. 2017:E1-E7.
18. Murayama R, Takahashi T, Tanabe H, Yabunaka K, Oe M, Oya M, et al. The relationship between the tip position of an indwelling venous catheter and the subcutaneous edema.

Biosci Trends. 2015;9:414-9.
19. Tanabe H, Takahashi T, Murayama R, Yabunaka K, Oe M, Matsui Y, et al. Using ultrasonography for vessel diameter assessment to prevent infiltration. J Infus Nurs. 2016;39:105-11.
20. Tanabe H, Murayama R, Yabunaka K, Oe M, Takahashi T, Komiyama C, et al. Low-angled peripheral intravenous catheter tip placement decreases phlebitis. J Vasc Access. 2016;17:542-7.
21. Maki DG, Ringer M. Risk factors for infusion-related phlebitis with small peripheral venous catheters. A randomized controlled trial. Ann Intern Med. 1991;114:845-54.
22. Stanley MD, Meister E, Fuschuber K. Infiltration during intravenous therapy in neonates: comparison of Teflon and Vialon catheters. South Med J. 1992;85:883-6.
23. J PT, D RA. The influence of the intravenous catheter composition on its hemocompatibility. PDA J Pharm Sci Technol. 1999;53:27-30.
24. Takashima M R-BG, Keogh S, Rickard CM. Randomised controlled trials in peripheral vascular access catheters: a scoping review. Vascular Access. 2015;1:10-37.
25. Resar R, Pronovost P, Haraden C, Simmonds T, Rainey T, Nolan T. Using a bundle approach to improve ventilator care processes and reduce ventilator-associated pneumonia. Jt Comm J Qual Patient Saf. 2005;31:243-8.
26. Burger CD, Resar RK. "Ventilator bundle" approach to prevention of ventilator-associated pneumonia. Mayo Clin Proc. 2006;81:849-50.

Study goals and objectives

This study aimed to establish and evaluate a three-point care bundle intervention method to prevent catheter failure.

Study Design

In this study, when examining the effect using RCT design in each catheter or patients, avoiding contamination among nurses working in the same department was difficult. Therefore, we adopted a non-randomized comparative non-blinding study design.

Methodology

Setting and recruitment

This study was conducted at the [name of institution] in Japan between July and

November 2017. The participants were recruited from two departments with high PIVC use, and no patients with extremely different attributes. Participants included patients >20 years who were hospitalized and received infusion therapy via a PIVC that was placed by nurses. Patients receiving chemotherapy and those with poor cognitive ability were excluded. The study procedures were explained to the doctors and nurses working in the ward at the beginning of the study period. Upon admission, patients who were expected to receive PIVC as part of their treatment were provided a written briefing of the study. Moreover, permission for patient intervention was obtained from the physician.

Intervention procedures

The definition of intervention was performing three items in the care bundle: "prescan for assessment vein diameter," "postscan for fixation according assessment catheter tip position in the vein," and "using polyurethane catheter." Intervention procedures were performed as follows. Prior to the data collection period, nurses received lectures and underwent briefing sessions for 30 min. The procedure for the intervention group for each catheter was as follows: a nurse planning to insert a PIVC assessed the site to be punctured and called a researcher; next, the researcher used US to assess vein size and location (prescan). Then, the researcher informed the result of the US examination to the nurse. Clinical nurses then decided the puncture point and performed the insertion. After the insertion, the researcher used US to observe and adjusted the catheter tip position in the vein (postscan).

The control group of patients was inserted with catheters using conventional care practices. The procedures for the control group were as follows: a nurse placed the PIVC and started intravenous therapy per routine practice with a Teflon® catheter according to implementation criteria in the hospital. Within 24 hours after cannulation, a researcher visually inspected the condition of the PIVC puncture site (using a photographic device, visually inspecting the site, and asking the patient about their pain) and used an US to measure vein diameter and depth.

Outcomes

The primary outcome was catheter failure, defined as the accidental and unplanned removal of catheters based on standard nursing practice for using PIVC in this hospital, according to the Centers for Disease Control and Prevention guidelines. Information regarding catheter failure was obtained from the medical chart and interviews. Catheter insertion and removal, as well as data regarding time, were described in

the medical charts at these wards. The researcher made direct observations at least twice a day and interviewed nurses and patients as much as possible. The researcher also collected dwell time (survival rate) with the occurrence of problems with catheter failure.

In this study, the researcher measured vein diameter and depth and assessed catheter tip position using US as surrogate outcomes. Vein diameter was defined as [major axis + minor axis]/2. The major axis was the longest diameter. The minor axis was perpendicular to the major axis. The major and minor axes were measured three times from the US image (0.04 mm/pixel). The mean of the three measurements was used for calculating the vein diameter. On identifying a target vein, the evaluators obtained a transverse image of the vein to measure the vein depth (distance from the skin surface to the superficial vein wall). The mean of three measurements was used for calculating vein depth. Appropriate catheter tip position was defined as the location in the lower side or at the center of the vein without an attached vein wall.

Other variables

The following data were collected by reviewing medical charts. Patient-related information included age, sex, body mass index (BMI), comorbidities (tumor or not at each organ), medical history (diabetes), and previous treatments (history of use of steroids, chemotherapeutic solutions, immunosuppressive solutions, anticoagulants, and radiation therapy); blood examination results included C-reactive protein (CRP), albumin, and platelet levels; administered medications at the baseline (use of hyperosmotic solutions, antibiotics, and fat emulsifiers); level of need for nursing (Kangodo [UMIN http://www.umin.ac.jp/kagoshima/]); and total time of locking. Nurse-related information included frequency of catheterization. The following data were collected through macroscopic observation: characteristics of the catheterization site position (anatomical insertion site, dominant vein, success of first catheterization attempt, catheterization times) and PIVC type (catheter material and size). The characteristics of the target vein (diameter and depth) and catheter tip position in the vein were determined using US examination according to the methods described in a previous study.

Data Management and Statistical Analysis

Sample size and study power

The setting was as follows: main analysis method, comparison of proportions; two-sided chi-square test; sample size, 356 catheters (178 catheters per group) with an effect

size of 0.15 (occurrence in the intervention group, 15%; occurrence in the control group, 30%), assuming a 10% dropout rate when setting α error = 0.05 and β error = 0.1 on power analysis. Data were collected over the same time period.

Statistics

Statistical analysis was performed using SPSS version 22.0 (IBM Corp., New York, NY, USA) and JMP Pro software version 13.0.0 (SAS Institute, Cary, NC, USA). A p value <0.05 was considered statistically significant.

The cumulative incidence rate of catheter failure during study periods, and the rate for each group per 1,000 device days as incidence rate (number of failures divided by the number of days catheterized then divided by 1,000) were calculated. We used Kaplan-Meier survival curves to compare rates of time until catheter failure between groups.

To minimize selection bias, we used inverse probability weighting (IPW) (32-34). Propensity scores were calculated using multiple logistic regression to predict the probability of each patient receiving intervention, using all subject characteristic, including age, sex, BMI, primary disease, disease history, therapy (steroids, chemotherapy, immunosuppressant, radiation therapy, anticoagulant), blood data (CRP, albumin, and platelet at baseline), catheterization times, medicine administration, level of need for nursing (Kangodo), and level of nurse experience in PIVC insertion. After propensity scores were calculated, intervention and control groups were balanced using IPW of propensity score.

Next, a multiple logistic regression analysis was used to estimate odds ratio (OR) and 95% confidence interval of factors contributing to catheter failure (n = 422). Variables with p values <0.05 in the univariate analysis were selected to be entered into hierarchical logistic regression analysis. Spearman rank correlation coefficients among the candidates for analyses were calculated for continuous variables. If coefficients >0.4 were found between the independent variables, only one variable was entered in the model. If one variable for multiple logistic regression was a categorical variable, then the t-test or the chi-square test was used. If the p value was <0.05, only one variable was entered. Age, sex, BMI, and performed intervention were selected as confounding factors.

Finally, subjects initially enrolled in the intervention group were included in the analysis (n = 270). Age, sex, BMI, anatomical site, and variables that were extracted from previous analysis were input as confounding variables, and the intervention contents added in this research, namely “catheter material” and “US assist,” were

entered for.

Project Management

Toshiaki Takahashi conceptualized and designed the study, designed the data collection instruments, collected data, coordinated and supervised data, carried out the initial analyses, drafted the initial manuscript and reviewed and revised the manuscript. Hiromi Sanada and Ryoko Murayama helped conceptualize and design the survey and critically revised the manuscript for important intellectual content. Miwa Nakamura, Mari Mizuno, and Chieko Komiyama designed the data collection instruments, collected data, carried out the initial analyses and critically revised the manuscript for important intellectual content. Mari Abe, Maki Miyahara and Chiho Kanno collected data and made substantial contribution to analysis and interpretation of the data, and critically revised the manuscript for important intellectual content.

Ethics, Informed Consent Forms

The study protocol was approved by the Ethics Committee of the Graduate School of Medicine, The University of Tokyo (approval #10707). Written informed consent was obtained from all residents or their proxies. The study was performed in accordance with the principles of the Declaration of Helsinki.
